# Distribution and source of plutonium in sediments from the southern Gulf of Mexico

**DOI:** 10.1007/s11356-022-18770-6

**Published:** 2022-01-25

**Authors:** José A. Corcho-Alvarado, Misael Díaz-Asencio, Stefan Röllin, Juan Carlos Herguera

**Affiliations:** 1 Nuclear Chemistry Division, Spiez Laboratory, Federal Office for Civil Protection, CH-3700 Spiez, Switzerland; 2 División de Oceanología, Centro de Investigación Científica y de Educación Superior de Ensenada (CICESE), Ensenada, Baja California México; 3 Escuela Nacional de Estudios Superiores (ENES), Unidad Mérida, UNAM. Mérida, Yucatán, México

**Keywords:** ^240^Pu/^239^Pu isotope ratio, ^239+240^Pu inventory, Deep-sea sediments, Nevada Test Site fallout, Global fallout

## Abstract

Here, we report on new data (75 analyses) of plutonium (Pu) isotopes to elucidate activity concentrations, inventories, sources, and their transport from the ocean surface to the seafloor from a collection of six deep-sea sediment cores (depths ranging from 257 to 3739 m) in the southern Gulf of Mexico. Sediment cores collected from the continental shelf and upper slope region of the Gulf of Mexico showed ^240^Pu/^239^Pu ratios of 0.15 to 0.26, and ^239+240^Pu-inventories ranging from 14.7 to 33.0 Bq m^−2^. Inventories and ratios are consistent with global fallout Pu for this tropical region. In contrast, sediment cores collected from the lower slope region and abyssal plain showed low ^240^Pu/^239^Pu ratios of 0.07 to 0.13 and much lower ^239+240^Pu inventories below 6.8 Bq m^−2^. This implies that only a small fraction of the expected global fallout Pu has reached the deep-sea sediments. The low ^240^Pu/^239^Pu isotope ratios indicate that fallout from the Nevada testing site was an important source of Pu in deep-sea sediments, and that this Pu was likely more efficiently scavenged from the water column than Pu from global fallout. We estimated that up to 44% of the total inventory of ^239+240^Pu in deep-sea sediments is due to the Nevada source. Low values and a progressive decrease of ^240^Pu/^239^Pu ratios and ^239+240^Pu inventories with increasing water depth have been previously reported for the Gulf of Mexico. Analysis of Pu isotopes in two sediment traps from the upper slope regions shows ^240^Pu/^239^Pu ratios comparable to those observed in global fallout. These results indicate that global fallout Pu is currently the main source of Pu in sinking particles in the water column. Therefore, a significant fraction of global fallout Pu must still be present, either in a dissolved phase, or as biologically recycled material in the water column, or scavenged on the shelf and shelf break. Our results bring to light important questions on the application of Pu isotopes to establish sediment chronologies in deep-sea sediments, since global fallout features such as the 1963 maximum are not available.

## Introduction

The ecosystems in the Gulf of Mexico are chronically exposed to hydrocarbons from natural seeps scattered throughout this region. In addition, the occurrence of major oil spills from anthropogenic activities, such as the Ixtoc spill in 1979 (Jernelöv and Lindén 1981, Schwing et al., 2021) or, more recently in 2010, the Deepwater Horizon (DWH) oil discharge (Joye et al. 2014, Brooks et al., 2015, Ziervogel et al. 2016), adds a several fold amount of hydrocarbons on an annual timescale. In the case of the DWH, a large though still not well-constrained fraction of the total oil released was deposited in the deep-sea environment (~ 1500 m) (Brooks et al., 2015), and surprisingly in a brief period of time after the accident (Joye et al. 2014, Brooks et al., 2015, Ziervogel et al. 2016, Schwing et al., 2021). Understanding the impact of hydrocarbons from natural seeps and oil spills in the northern region of the Gulf of Mexico (shelf and upper slope environments) has increased in the last decade. This is a result of different scientific research programs like the Gulf of Mexico Alliance (https://gulfofmexicoalliance.org/) or the Gulf of Mexico Research Initiative (GOMRI, https://gulfresearchinitiative.org/). However, research in the southern region of the Gulf of Mexico is still limited.

Proxy records recovered from sediments provide time series that may reveal clues to better understand the impact of past events. Obtaining proxy data from sedimentary records relies mainly on radiometric dating methods (e.g., ^14^C and ^210^Pb) (Yeager et al. 2004, Corcho-Alvarado et al. 2014, Foucher et al. 2021, Li, Li et al. 2021). For recent sediments (past 100 years), chronologies obtained using ^210^Pb have shown to be reliable in high sediment accumulation environments (Appleby and Oldfield 1978; Appleby and Oldfieldz 1983). However, the ^210^Pb ages may suffer from different biases and usually need to be verified using an independent method (Appleby 1998; Santschi and Rowe 2008; Corcho-Alvarado et al. 2014, Barsanti et al., 2020). For this purpose, other chronostratigraphic markers such as the global fallout radionuclides (e.g., ^137^Cs, ^239+240^Pu, and ^241^Am) are commonly measured in sedimentary records (Appleby et al. 1986, Appleby et al. 1991, Lindahl et al. 2010, Díaz-Asencio et al. 2016, Díaz-Asencio et al., 2020c, Foucher et al. 2021). Global fallout radionuclides are characterized by a distinct maximum in 1963, which is related to the period of higher intensity of nuclear weapons testing (Corcho-Alvarado et al. 2014; Foucher et al. 2021). This fallout maximum (peak) can be identified in sedimentary records and further used as a time marker. Among the fallout radionuclides, plutonium (Pu) isotopes have found useful application in the Gulf of Mexico region (Scott et al. 1983; Buesseler and Sholkovitz 1987a, b; Buesseler and Sholkovitz 1987a, b; Oktay et al. 2000; Yeager et al. 2004; Corcho-Alvarado et al. 2014). ^137^Cs, another commonly used chronostratigraphic marker, is present at low or negligible levels in deep-sea sediments of the Gulf of Mexico (Yeager et al. 2004; Corcho-Alvarado et al. 2014; Carnero-Bravo et al. 2018, Díaz-Asencio et al., 2020a).

Pu isotopes in the southern Gulf of Mexico have two well-defined sources, global atmospheric fallout from nuclear weapon testing (direct fallout or continental runoff) and regional fallout from nuclear weapon testing in Nevada (USA) (Scott et al. 1983; Buesseler and Sholkovitz 1987a, b; Oktay et al. 2000). These two sources have distinct isotopic signatures and fallout characteristics that differentiate them. The integrated worldwide global fallout of ^240^Pu/^239^Pu isotope ratio has a mean value of 0.180 ± 0.014 (Kelley et al. 1999a, 1999b), in contrast to the regional derived Pu fallout from the Nevada Test Site (NTS) that shows much lower mean ratios close to 0.032 ± 0.003 (Hicks and Barr 1984; Buesseler and Sholkovitz 1987a, b). Moreover, while global fallout is carried by small size particles of 1 µm or less (Joseph et al., 1971), NTS fallout is borne by larger particles of 1 to 100 µm diameter (Joseph et al., 1971). The particle size distribution and the chemical/physical form of the Pu-bearing fallout particles have important implications for the Pu geochemistry in the ocean and their transport to and in the ocean (Buesseler and Sholkovitz 1987a, b; Buesseler and Sholkovitz 1987a, b; Buesseler 1997; Povinec et al. 2003; Hamilton 2005). Scott et al (1983) first hypothesized that material from the NTS was transported through the troposphere to the Gulf of Mexico, where Pu was scavenged to the sediments more efficiently than other components of Pu fallout. Buesseler and Sholkovitz (1987a, b) later showed that particles from the NTS fallout were rapidly removed to the sediments, accounting for over 40% of the Pu inventory in the Northwestern Atlantic. Global Pu fallout, on the other hand, accounted for most of the Pu in the water column and in shallow coastal sediments. Although these studies have shed some light on the fate of the Pu isotopes in the deep sediments of the Gulf of Mexico, the information is still limited.

This study focuses on further investigating the source of Pu in deep sediments of the Gulf of Mexico and its main transport processes. The main objectives of the study were (i) to measure the Pu activities and ^240^Pu/^239^Pu atom ratios in sediment profiles and particle traps from the Gulf of Mexico; (ii) to evaluate the relative contributions of the global stratospheric fallout Pu and the regional tropospheric fallout Pu from the NTS to the deep-sea sediments of the Gulf of Mexico using a two-end-member mixing model; and (iii) to propose a transport pathway of Pu fallout to the Gulf of Mexico. Another important objective of this work is to define the input function of Pu to deep-sea sediments and to verify if Pu radionuclides can be used as a time marker for sedimentation studies. This information is especially important for other investigations being carried out in parallel in the Gulf of Mexico, which are aimed at investigating the accumulation, degradation, and burial of organic matter, hydrocarbons, and other contaminants in deep-sea regions. The results have also worldwide implications, particularly when investigating deep-sea sediments.

## Methods

### Study location

**Figure 1.**
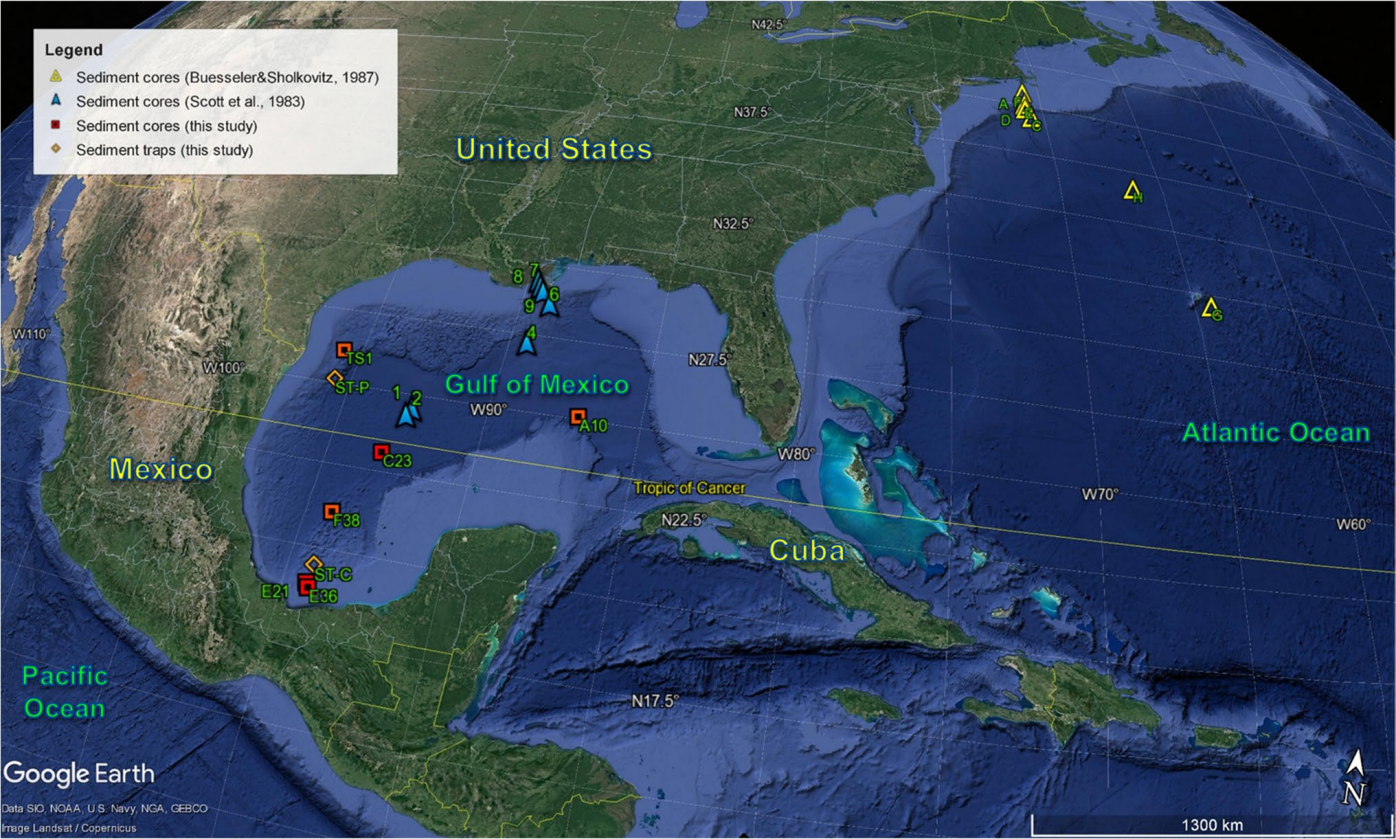
Location of the sediment cores (red squares) and sediment traps (orange rhombus) investigated in this study. The sites investigated by Scott et al. (1983) (blue triangles) in the Gulf of Mexico, and by Buesseler and Sholkovitz (1987a, b) (yellow circle) in the Western North Atlantic are also shown. Map was created in Google Earth.

The Gulf of Mexico is a semi-enclosed oceanic basin flanked by two wide carbonate shelfs, located between the tropics and subtropics in the western Atlantic Ocean (Fig. 1). The region of our study in the southern Gulf of Mexico extends between the Sigsbee Abyssal Plain to the north, the Yucatan Shelf and Campeche Escarpment to the northeast and east, the Tamaulipas and Veracruz continental slopes to the west, and by the Campeche Saline Complex to the south (Fig. 1). The Florida and Campeche shelfs contribute with carbonate, and essentially very little siliciclastic sediments to the basin (Holmes 1976). Siliciclastic sediments input is mostly associated to several river systems, Mississippi-Atchafalaya system (United States) in the northern region, rivers carving the Eastern Sierra Madre (Mexico) to the west, and the Grijalva-Usumacinta river (Guatemala and Mexico) complex to the south. Most of these sediments are deposited on the continental shelf, and a fraction escapes the continental platform environments depositing on the continental slopes and an even smaller fraction reaches the abyssal plain (Brooks et al., 2015, Díaz-Asencio et al. 2019). Sediments beyond the shelf edge show a decreasing trend in the terrigenous components between the slopes and the abyssal plain (Morse and Beazley 2008; Díaz-Asencio et al. 2019).

### Sampling

Two sediment cores collected in 2018 from the upper continental slope in the Campeche Saline Complex, and four sediment cores collected in 2019 from the lower continental slopes and the abyssal plain of the Gulf of Mexico are used in this study (see Table 1 and Fig. 1). The six sediment cores were retrieved using a Soutar box core (40 × 40 cm), from which cores were subsampled on board using acrylic tubes (Internal diameter of 10 cm). Core lengths generally varied between 20 and 30 cm (Table 1). Sediment cores were split along their depth axes, one-half was sectioned at 0.5 cm for geochemical and radionuclides analysis. Sediment samples were then frozen and later freeze-dried.

**Table 1.** Total ^239+240^Pu inventory and mean ^240^Pu/^239^Pu isotope ratio in sediment cores from the southern Gulf of Mexico. Information about the sediment traps are also reported. The percentage of ^239+240^Pu delivered to each site by regional fallout from the Nevada Testing Site is also shown. For comparison, results from previous studies by Scott et al. (1983) in the same region, and Buesseler and Sholkovitz (1987a, b) in the North West Atlantic, are also presented. Expanded uncertainties with *k*=2 are reported. For the error propagation, an uncertainty of 5% was assumed for the total ^239+240^Pu inventories from previous studies

Site	Depth	Location		Core length	^240^Pu/^239^Pu isotope ratio	Total ^239+240^Pu inventory	NTS ^239+240^Pu	Global fallout ^239+240^Pu
	(m)	Lat.	Long.	(cm)		(Bq m^−2^)	(%)	(Bq m^−2^)
Gulf of Mexico (This study)								
E36	257	18°46.1′N	94°1.4′W	20	0.16 ± 0.01	33.0 ± 0.9	8 ± 2	30 ± 9
E21	419	18°53.8′N	94°7.0′W	20	0.17 ± 0.01	14.7 ± 0.5	3 ± 1	14 ± 5
TS1	2417	26°0.6′N	95°35.5′W	24	0.10 ± 0.02	6.8 ± 0.3	42 ± 21	4 ± 2
F38	2822	21°0.5′N	94°0.2′W	26	0.11 ± 0.03	4.8 ± 0.2	36 ± 21	3 ± 2
A10	3340	24°59.4′N	86°59.0′W	27	0.14 ± 0.04	3.3 ± 0.2	21 ± 14	2.6 ± 1.8
C23	3739	22°59.9′N	93°0.2′W	24	0.10 ± 0.02	1.3 ± 0.1	44 ± 21	0.7 ± 0.3
Gulf of Mexico (Scott et al. 1983)								
Station 9	106	28°44.0′N	89°25.9′W		0.183 ± 0.002	410	< 0	410
Station 8	320	28°32.1′N	89°17.7′W		0.172 ± 0.003	232	4 ± 1	223 ± 56
Station 7	786	28°21.6′N	89°09.0′W		0.141 ± 0.005	13	19 ± 50	11 ± 3
Station 6	1701	27°57.5′N	88°47.7′W		0.138 ± 0.010	5	21 ± 6	4 ± 1
Station 4	2744	26°34.0′N	89°11.3′W		0.106 ± 0.001	7	41 ± 10	4 ± 1
Station 1	3402	23°43.9′N	92°28.0′W		0.10 ± 0.02	5	43 ± 21	3 ± 1
Station 2	3649	23°57.1′N	92°19.9′W		0.10 ± 0.02	5	44 ± 22	3 ± 1
North West Atlantic (Buesseler and Sholkovitz 1987a, b)								
A	90	40°28.1′N	70°54.1′W		0.19 ± 0.02	207	< 0	207
F	501	39°55.1′N	70°54.1′W		0.18 ± 0.01	44	2 ± 1	43 ± 12
E	1275	39°48.1′N	70°56.3′W		0.152 ± 0.006	38	14 ± 4	33 ± 9
D	2362	39°35.0′N	70°56.8′W		0.137 ± 0.007	21	22 ± 6	17 ± 4
C	2700	39°10.3′N	70°43.8′W		0.13 ± 0.01	5	28 ± 9	3 ± 1
G	4469	31°54.1′N	64°17.8′W		0.10 ± 0.01	4	43 ± 15	2 ± 1
H	4990	36°27.9′N	66°33.6′W		0.10 ± 0.01	7	41 ± 10	4 ± 1
Sediment traps (This study)								
ST-P	1000	25°0.5′N	95°32.3′W	-	0.17 ± 0.02	Perdidos region (water depth: 1130 m)		
ST-C	1000	19°23.6′N	94°3.8′W	-	0.17 ± 0.01	Coatzacoalcos region (water depth: 1100 m)		

Two sediment traps (Parflux Mark78H-21, with a catchment area of 0.5 m^2^ and 21 collection cups of 0.5 L) were deployed in the western and southern slopes of the Gulf of Mexico (see Table 1 and Fig. 1). One of the traps was located in the western slope of the Gulf of Mexico (Perdido region, ST-P), relatively close to land (ca.114 km) and at a water depth of 1130 m (55 m above the seafloor sediments). The other trap was located in the Campeche Saline Complex slope (Coatzacoalcos region, ST-C), also relatively close to land (ca. 120 km) and at a water depth of 1100 m (55 m above the seafloor). This region is located close to the Campeche Bay quasi-permanent cyclonic eddies (Pérez-Brunius et al. 2018) and periodically blanketed by sediments from the Grijalva-Usumacinta River system (Guatemala and Mexico). Before deployment, each collection cup was filled with a hypersaline seawater solution prepared with filtered seawater, Na_2_B_4_O_7_, NaCl, and formaldehyde at 37% (to retard bacterial activity in the trap material). The period of collection for each cups in both traps lasted for 18 days. After sediment traps were recovered, samples were sieved through a 1000 μm nylon screen to remove swimmers and large aggregates.

### Analyses

Sediment samples were analyzed for Pu radioisotopes as described elsewhere (Röllin et al. 2009, Sahli et al., 2017, Röllin et al., 2020). Briefly, about 5 g aliquots were spiked with known amounts of ^242^Pu (about 3 pg). The aliquots were then digested by a borate fusion using 50 mL Pt/Au (95%/5%) crucibles in a furnace at 1100 °C. The melt was poured into 4.5 M HNO_3_ and the silicates were precipitated with Poly-Ethylene Glycol (PEG) (Röllin et al. 2009). After filtration, Pu was separated from other radionuclides in a TEVA resin as described elsewhere (Sahli et al. 2017). Pu radioisotopes were analyzed with a double-focusing magnetic sector field inductively coupled plasma mass spectrometer (SF-ICP-MS) Element XR (Thermo Fischer Scientific) (Röllin et al. 2009). An Apex nebulizing system connected to an ACM desolvator and a self-aspirating PFA-ST-nebuliser (all from Elemental Scientific Incorporation, USA) were used for introducing the samples into the system. Pu isotope concentrations were calculated from the signal of the ^242^Pu tracer. The contributions of the Pu isotopes from the tracer and tailing from U and Th were corrected mathematically based on the isotope ratios from the certificate and abundance sensitivity measurements of U and Th standards. The mean radiochemical yield of Pu was 95 ± 7 %. The certified reference materials IAEA-135 (Irish Sea sediment) and IAEA-384 (Fangataufa Lagoon sediment) obtained from the International Atomic Energy Agency (IAEA, Vienna, Austria) were used for testing the analytical method. Uncertainties were calculated by standard propagation of all experimental uncertainty sources, including 1 sigma counting errors of samples and blanks.

### Data interpretation

We use a two-end-member mixing model to estimate the relative contribution of each Pu fallout source to the sediments in the Gulf of Mexico (Krey et al., 1976). The model is based on the distinct ^240^Pu/^239^Pu isotope ratios of the two sources of Pu: (a) global fallout (GF) with a ratio of 0.180 ± 0.014 (Kelley et al. 1999a, 1999b) and (b) regional tropospheric fallout from Nevada (NTS) with a ratio of 0.032 ± 0.003 (Hicks and Barr 1984). The percentage of Nevada Pu fallout (*%NTS*) in the sediments can be derived from the following equations:

1 $$A= \frac{{Pu}_{NTS}}{{Pu}_{GF}}=\left(\frac{{R}_{GF}-{R}_{sample}}{{R}_{sample}-{R}_{NTS}}\right)\bullet \frac{\left(1+3.673\bullet {R}_{NTS}\right)}{\left(1+3.673\bullet {R}_{GF}\right)}$$

2 $$\%NTS=100\bullet \left(\frac{A}{1+A}\right)$$

where *R*_*sample*_, *R*_*GF*_, and *R*_*NTS*_ are the ^240^Pu/^239^Pu isotope ratios in the sample (measured), global fallout (0.18) and the NTS (0.032), respectively, and 3.673 is a factor for conversion between atom and activity ratios of ^240^Pu/^239^Pu. The uncertainties are propagated from the analytical uncertainties (expanded uncertainty *k* = 2) and uncertainties reported for the two fallout end-members, global fallout, and Nevada fallout.

## Results and discussion

**Figure 2.**
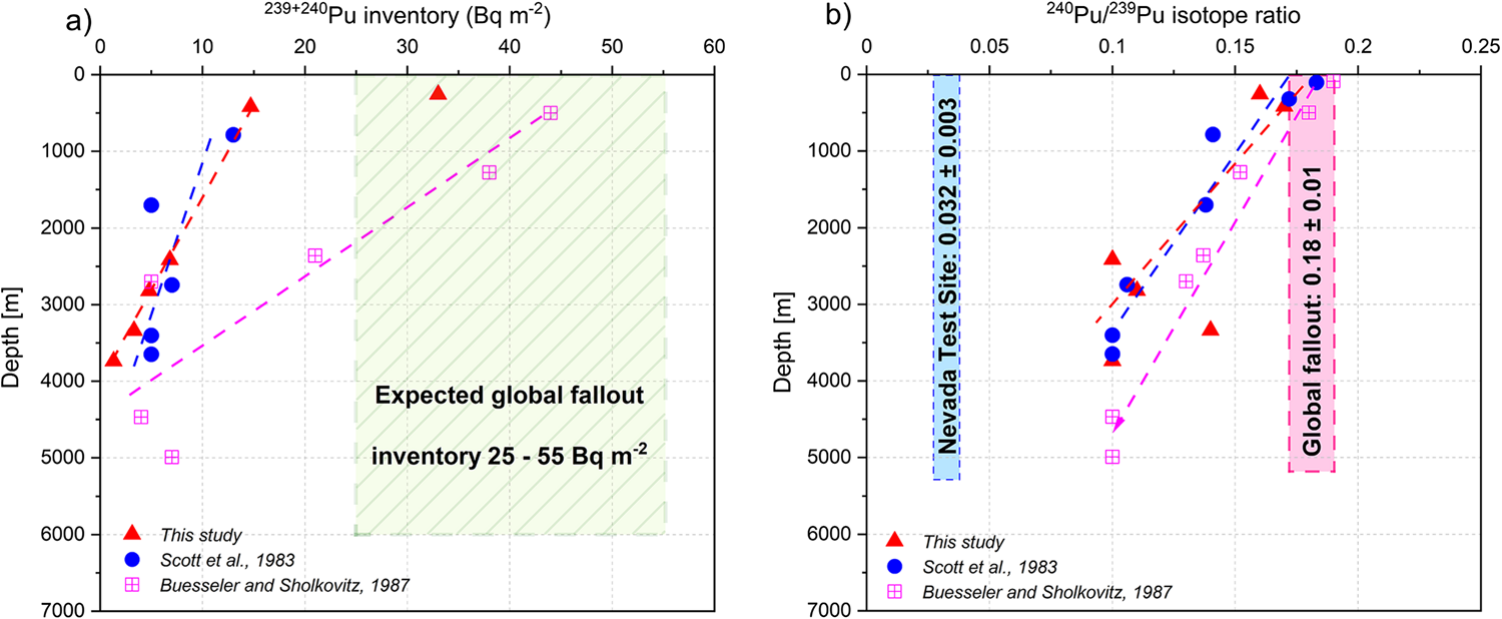
Relationship of the a) ^239+240^Pu inventory and b) mean ^240^Pu/^239^Pu isotope ratio with the depth of the sampling site in the southern Gulf of Mexico. For comparison, the results reported by Scott et al. (1983) for the Gulf of Mexico, and the ones from Buesseler and Sholkovitz (1987a, b) for the Western North Atlantic are also shown.

The plutonium analysis for sites E36, E21, TS1, F38, A10, and C23 is given in Table 1. The percentage of ^239+240^Pu delivered to each site by regional fallout from the NTS was calculated according to Equation (1) and is listed in Table 1 along with the measured ^239+240^Pu inventory for each core. Sites located in the continental shelf and upper continental slope showed ^239+240^Pu inventories (14.7 to 33.0 Bq m^−2^; Table 1 and Fig. 2a) that are in the same order of magnitude to those expected from global fallout (25–55 Bq m^−2^) at these latitudes (Corcho-Alvarado et al. 2014). However, sediment cores collected from the abyssal plain and lower continental slope showed significantly lower ^239,240^Pu inventories (<6.8 Bq m^−2^) (Fig. 2a). These deep-sea sites contain only a small fraction (0.7 to 3.9﻿ Bq m^−2^; Table 1) of the expected ^239,240^Pu inventory from global fallout. These results are consistent with those reported by Scott et al (1983) and Yeager et al (2004), who observed as well low ^239+240^Pu inventories in abyssal sediments in the Gulf of Mexico (Scott et al. 1983; Yeager et al. 2004). In deep-sea sediments, only a fraction of the global fallout Pu delivered to the ocean surface has been generally found (Scott et al. 1983; Li et al. 1985; Nagaya and Nakamura 1993; Garcia-Orellana et al. 2009; Kinoshita et al. 2011). Low Pu inventories of about 20% of the total global fallout Pu (approximately 8.9 Bq m^−2^ according to (Hardy et al. 1973)) were reported in abyssal sediments from the Venezuela Basin, in the Caribbean Sea (Li et al. 1985). Similarly, Pu inventories (4 to 44 Bq m^−2^; Table 1) accounting for only 4 to 50% of that expected from global fallout (approximately 93 Bq m^−2^ according to (Hardy et al. 1973)) were estimated in deep sediments collected between 1983 and 1985 in the North West Atlantic (Buesseler and Sholkovitz 1987a, b). The low Pu inventories in deep-sea sediments of the southern Gulf of Mexico are thus consistent with values previously published in comparable zones.

It should be noted that the Pu inventories of 1.3 to 14.7 Bq m^−2^ observed in the abyssal and continental slope sediments are comparable to those of 5 to 13 Bq m^−2^ reported by Scott et al. (1982), about 40 years earlier (Table 1 and Fig. 2a). This is a strong indication that Pu transport from ocean water to deep-sea sediments over the past four decades has been very low or negligible. Therefore, Pu currently present in deep-sea sediments was most likely scavenged in a relatively short period after its deposition at the ocean surface. This points out that both Pu fallout components, NTS and global fallout, entered the deep-sea sediments possible as a pulse-like function, and not continuously, as it is commonly observed in shallow coastal sites (Corcho-Alvarado et al. 2014; Carnero-Bravo et al. 2016, 2018). This is further supported by the limited variability (less than 17%) of the ^240^Pu/^239^Pu isotope ratios within each core (Table 1).

A large fraction of global fallout Pu is likely in the water column (e.g., subject to biogeochemical cycling Povinec et al. 2003; Hamilton 2005)) and/or has been horizontally transported to shallower sites where it has been scavenged to the sediments (Scott et al. 1983). According to Hamilton (1995), about 80% of the total ^239+240^Pu inventory in the open ocean in 2005 was still in the water column. Numerous studies have shown that global fallout Pu is mostly found in the ocean water column, concentrated at a given depth below the surface (Bowen et al. 1980; Buesseler 1997; Hirose et al. 2011; Kinoshita et al. 2011, Rozmaric et al., 2021). In the Pacific Ocean, a pronounced subsurface maximum of Pu concentrations was first observed at depths varying between 250 to 750 m during a GEOSECS study in 1973/1974 (Bowen et al. 1980). Other studies in central south and tropical east Pacific reported as well subsurface Pu maximums at depths of 500 to 800 m (Povinec et al. 2003; Hirose et al. 2011; Kinoshita et al. 2011). In the northwest Pacific Ocean, time series of Pu in the water column obtained over 24 years showed that the Pu subsurface maximum was moving downward (from 450 to 850 m) and getting smaller and less pronounced (a decrease by about a factor 4) (Livingston et al. 2001; Povinec et al. 2003). In a recent study in the tropical East Pacific, Kinoshita et al. (2011) demonstrated that the Pu subsurface maximum was indeed slowly moving downward at mean velocities of 3 to 17 m year^−1^ (Kinoshita et al. 2011). A subsurface Pu maximum has been observed in other regions of the Pacific such as in the South China Sea and the Sulu Sea (Dong et al. 2010), the Sea of Japan (Hirose et al. 2002), and in other oceans such as in the Mediterranean Sea (Vintró et al. 1999; Fowler et al. 2000; Laissaoui et al. 2008), the northwest Atlantic (Cochran et al. 1987), or the Benguela upwelling system in the southeast Atlantic (Rozmaric et al., 2021).

In deep-sea environments, Pu transport to bottom waters does occur. However, as deep zones do not receive significant particulate inputs from the shelf, there is typically a low density of sinking particles (Vintró et al., 2005). Sedimentation rate at such deep sites is extremely low in the order of a few cm per 1000 years (Garcia-Orellana et al. 2009; Carvalho et al. 2011). Hence, Pu levels in deep-sea sediment are rather low (Li et al. 1985; Nagaya and Nakamura 1993; Garcia-Orellana et al. 2009). Inventories of Pu in deep-sea sediments in the tropical east Pacific were up to 15 times lower than those found in the water column (Kinoshita et al. 2011). In another study in the northern North Pacific Ocean, ^239,240^Pu inventories in deep sediments were less than 20% of those found in the entire water column (Nagaya and Nakamura 1993). In the Mediterranean Sea, less than 3% of the global fallout Pu deposited across the sea surface was found in deep sediments (Garcia-Orellana et al. 2009). Similarly, in the Venezuelan Basin (Caribbean Sea), sediments (depth: 3500 to 5050 m) contained approximately 20% of the total Pu that has fallen to the ocean surface. All these studies evidence that a large fraction of global fallout Pu is still in the water column and supported the existence of a subsurface maximum of Pu activity concentrations in most deep sites of our oceans. Unfortunately, no vertical profile of Pu in ocean water is available for the Gulf of Mexico. It is nonetheless not ruled out that such a subsurface Pu maximum is present in the water column of this gulf, as it has been suggested elsewhere (Scott et al. 1983).

**Figure 3.**
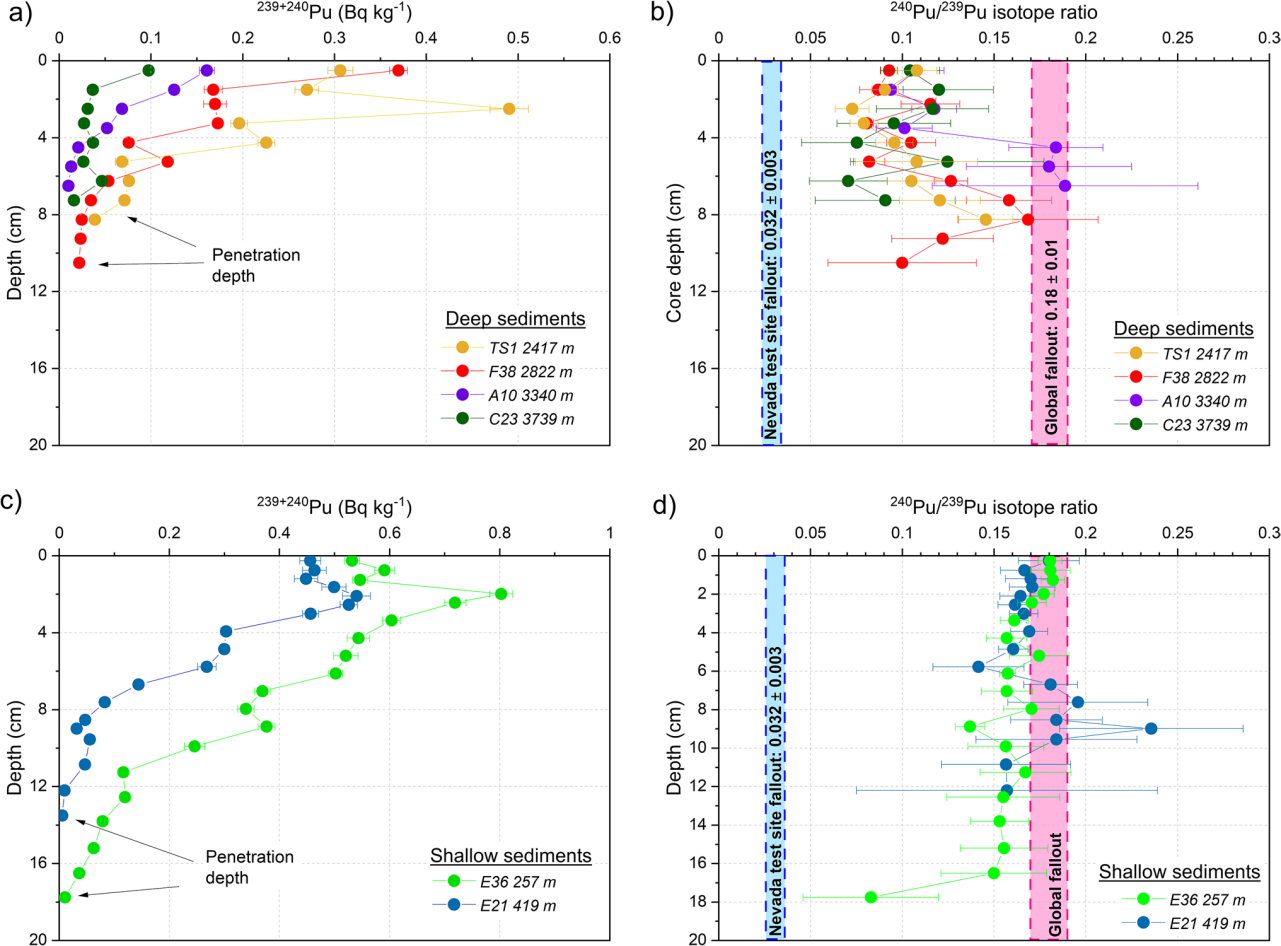
Profiles of ^239+240^Pu activity concentrations and ^240^Pu/^239^Pu isotope ratios in the sediment cores collected in the southern Gulf of Mexico. In order to gain visibility, shallow and deep cores are plotted in separate graphs. Expanded uncertainties with *k*=2 are reported.

The ^239+240^Pu activity concentration and ^240^Pu/^239^Pu isotope ratio versus depth within each sediment core are plotted in Figure 3. A common feature in all the investigated sites is that Pu penetration depth is found below 6 cm, which is deeper than it would be expected from the low sedimentation accumulation rates of a few cm per 1000 years reported for this region (Díaz-Asencio et al., 2020b). In cores from the abyssal plain and lower slope, Pu isotopes are found at 6 to 11 cm depth (Fig. 3a), while in the upper slope and continental shelf, they are found slightly deeper at 12 to 19 cm depth (Fig. 3c). This is a strong indication that Pu profiles are primarily a result of mixing likely by bioturbation on the seafloor. This process is as well responsible for the little variability of the ^240^Pu/^239^Pu isotope ratio with depth within each core (Fig. 3b, d). Previous studies have shown the importance of bioturbation by benthic in deep-sea sediments of the Gulf of Mexico (Scott et al. 1983; Yeager et al. 2004; Santschi and Rowe 2008, Díaz-Asencio, Herguera et al. 2020). Bioturbation is likely responsible for the not well-defined peaks of ^239+240^Pu observed at approximately 2 to 3 cm depth in the cores E36, E21, and TS1 (Fig. 3a, c). These peaks are certainly not related to the global fallout maximum of 1963. The implication of this finding is that Pu in deep-sea sediments of the Gulf of Mexico is possibly a tracer of bioturbation rather than sediment accumulation.

Based on the little variability of the ^240^Pu/^239^Pu ratio in the upper 8 cm of the sediment cores, a mean isotope ratio was calculated for each site and is reported in Table 1. The mean ^240^Pu/^239^Pu ratio showed a consistent decrease with increasing water depths, from a ratio of 0.16 to 0.17, at shallow depths, to ratios close to 0.10 below 2000 m (Fig. 2a). These results are comparable to those previously reported from deep sediments of the Gulf of Mexico (Scott et al. 1983) and the North West Atlantic (Buesseler and Sholkovitz 1987a, b).

Using Equations (1) and (2), we estimated the percentage of NTS fallout Pu in each site. The results of these calculations are shown in Table 1. The percentage of Nevada-derived Pu is higher in the abyssal and lower continental slope sediments (21 to 44 %) than in the upper continental slope and shelf sediments (3 to 8 %). The decreasing trend of ^240^Pu/^239^Pu isotope ratios from 0.17 to 0.10 with increasing depths from 257 to 3739 m (Fig. 2b) implies a lower contribution of global fallout Pu at any given site in respect to the more rapidly deposited NTS fallout. Previous studies have shown that regional tropospheric fallout Pu from surface nuclear weapon tests is scavenged to the sediments more effectively than global fallout Pu (Buesseler and Sholkovitz 1987a, b; Buesseler 1997; Povinec et al. 2003; Dong et al. 2010). In shallow water sediments close to the lithogenic input, the high particle loads favor an enhanced Pu scavenging, and consequently, the ^240^Pu/^239^Pu isotope ratio is controlled by the global fallout input (^240^Pu/^239^Pu ratio close to 0.180 ± 0.014; Fig. 2b). In contrast to deep-sea waters where much lower particle loads control a low Pu scavenging (Scott et al. 1983; Buesseler and Sholkovitz 1987a, b) and explain the lower NTS fallout signature (^240^Pu/^239^Pu ratio close to 0.10; Fig. 2b).

**Figure 4.**
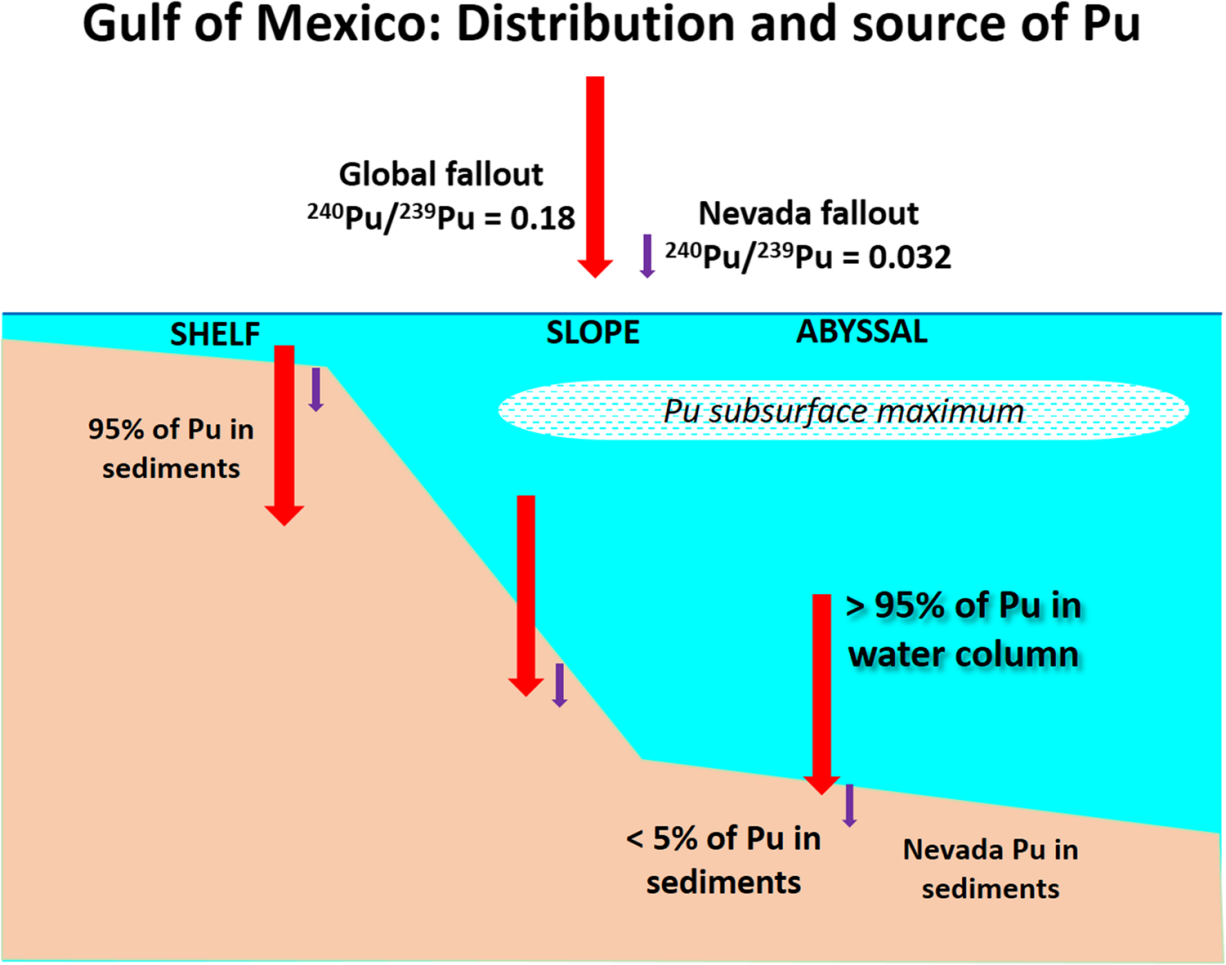
Conceptual model of the Pu sources and distribution in the Gulf of Mexico (Model after (Buesseler and Sholkovitz 1987a, b)).

Results from particle traps deployed in the Perdido region and the Campeche Saline Complex show mean vertical particle fluxes in the water column of 105 and 119 g m^−2^ year^−1^, respectively. These particle fluxes fall within the range of those reported in the northern Gulf of Mexico (35 to 175 g m^−2^ year^−1^) (Giering et al., 2018). A mean ^240^Pu/^239^Pu isotope ratio of 0.17 ± 0.01 (Table 1), consistent with the ratio observed in global fallout, was measured in bulk dry material collected at these two traps. This implies that global fallout is the main source of Pu in the water column of the Gulf of Mexico (Fig. 4).

The observed range of ^240^Pu/^239^Pu isotope ratios of 0.09 to 0.11 in deep-sea sediments (> 2000 m) is comparable to those reported by Scott et al. (0.10 to 0.14, (Scott et al. 1983)), about four decades earlier. Considering that Pu in the water column currently shows a ^240^Pu/^239^Pu ratio of 0.17± 0.01, the remarkably similar ratios of these two studies separated by 40 years argue against an additional deposition of global fallout Pu over the past four decades at these sites.

Our results further imply that Pu in deep-sea sediments of the Gulf of Mexico have entered as a pulse-like function, and not continuously as it is usually observed in shallow sea sediments. Buesseler and Sholkovitz (1987a, b) were the first to propose such an input in their interpretation of Pu isotopes in deep-sea sediments from the Northwestern Atlantic Ocean (Buesseler and Sholkovitz 1987a, b). According to this model, significant inputs of regional fallout Pu from the NTS would be expected during the 1950s, when the most intensive atmospheric testing took place in Nevada. The second source of Pu, global fallout, started in the 1950s, had a maximum in 1963 and then decreased rapidly. For this source, a pulse input during 1960 can be assumed. These assumptions are in agreement with the findings from Oktay et al. (2004), who showed that Pu fallout from the NTS reached the Mississippi River Delta sediments during the early 1950s, while the global fallout maximum was identified at a shallower depth within the investigated core (Oktay et al. 2000). These results have important implications for interpreting Pu isotopes in deep-sea sediment and for investigating the transport of other contaminants such as large oil spills.

## Conclusions

This study shows that Pu isotopes in deep-sea sediments in the Gulf of Mexico originated from two separate sources, on the one hand the regional fallout from the NTS and on the other the global stratospheric fallout. The results here reported on the distinct ^240^Pu/^239^Pu isotope ratio of these sources show that the relative contribution of the NTS to the total Pu in the sediments increases with depth, accounting for over 45% in the abyssal region. We do not find any significant deposition of Pu over the past four decades. These results further evidence that a pulse-like input of Pu to the sediments is the most likely approximation for deep-sea sediments. Moreover, our results further support the model proposed by Scott et al (1983) for the deposition and transport of Pu in the Gulf of Mexico, implying that the vast majority of global fallout Pu still remains in the water column. The analysis of the ^240^Pu/^239^Pu isotope ratio in samples collected from two particle traps, located at about 1000 m depth, confirmed that global fallout is the largest source of Pu in the water column. Another implication of these results is the potential of Pu isotopes as tracers of bioturbation mixing rather than accumulation processes in deep-sea sediments.

## Data Availability

All data generated or analyzed during this study are included in this published article
